# Notes and News

**Published:** 1894-06-30

**Authors:** 


### Notes and News.

Collected and Communicated.

The Hospital Sunday Fund collection for this year has now reached £35,500.

The prize offered by the King of Italy for research in experimental pathology and physiology has been divided by the Accademia dei Lincei of Rome between Professor Q. Tizzoni, of Bologna, and Professor Luciani, of Rome.

Public feeling seems inclined to lead the way for legislation in the matter of small-pox infection in America. It is stated that a negro has been lynched who was suffering from the disease, and in whose case proper precautions were not taken; and it is also stated that a Chicago policeman actually asked permission to shoot a delirious small-pox patient.

A new wing has just been added to the Cookridge Convalescent Hospital for Leeds. This wing has been built in memory of the Rev. Canon Jackson, he in the course of his philanthropic life having taken much interest in the institution. The extension will give accommodation to a further fifteen patients; it consists of two dormitories, dining, day, and recreation rooms. £1,000 is yet required to complete the furnishing, £3,000 having so far been expended.

The small Convalescent Home for Children at Weston-super-Mare, founded by Miss Cornish, has also undergone extension. The establishment has been moved by Mrs. Hutchinson, of Clifton, into larger premises, and much care has been exercised to render the home complete. One of the features is a large playroom, thirty-eight feet by twenty-one feet, which will prove a source of happiness to the young inmates.

It would seem that we are to court indigestion for the sake of our health. Whilst time-honoured domestic precepts have warned us against the dangers of new bread, a Russian medical man, Dr. Troitzki, puts forward the advantage of new bread by showing that it contains no micro-organisms, the heat being sufficient to kill them. When the bread is once cut and left about, we are told that it forms an excellent refuge for microbes. Those who would face anything rather than microbes must make up their minds to face indigestion or eschew bread.

The fête at the British Home for Incurables, which will be opened on July 3rd by the Princess of Wales, promises to be a brilliant affair. A number of well-known ladies will take charge of the various booths and kiosks to be placed in the grounds. Several of these booths will be devoted to entertainments and exhibitions. Sir Augustus Harris will organise and superintend the theatrical features, and numbers of well-known artistes have promised their services.

Sir Algernon Borthwick is an ideal chairman for a festival dinner. His cheery and genuine interest in the whole of the proceedings, his happy gift of eloquent expression, and the trouble he takes to really master the details of the work for which the chairman has to appeal were very noticeable at the banquet for the Chelsea Hospital for Women. Sir Algernon Borthwick has something of the look of Lord Salisbury about him, and no one who was present on Tuesday could fail to be impressed with his fearless loyalty to the best interests of an institution to which he has once given his name. These are rare qualities which will no doubt tend to make the proprietor of the *Morning Post* one of the most popular of presidents, as he is undoubtedly and deservedly one of the most popular of men.

Each Scotch medical school in its turn seems destined to become the battlefield upon which female medical students have at the outset to meet repulse. The Dundee Royal Infirmary is the last centre of medical education asked to open its doors to lady students. The application has met with definite refusal. The two strongest reasons urged against it were, firstly, that there was no precedent, and secondly, that such an innovation would tend to make the infirmary less capable of curing diseases. Of all reasons to bring forward these were of the poorest, and Edinburgh could indignantly repudiate the last were not the fame and popularity of its Royal Infirmary too widespread to need any vindication. The inhabitants of Dundee do not as a whole appear to sympathise with the obstructive policy of the managers of the infirmary.

The care and protection of young children is an especial feature of the civilisation of our age. In England we provide for and enforce that neglect or cruelty shall be as little present amongst the children of the poor as is possible. The Society for the Prevention of Cruelty to Children is actively at work, and is ever ready to increase its sphere of action. Its salutary effects are recognised over the Continent, and now in Paris the authorities of the Assistance Publique have arranged that Dr. Henri Jolly shall come and study the methods of the society. Dr. Jolly has been engaged in studying the treatment of children, and the opportunities of protection afforded them throughout Europe, and it is to be confidently hoped that England and her efforts will come out, if last, certainly not least to be commended on the list. In the mountainous region of the Vosges, through the energies of Dr. Brallet, a medical inspection is made of the condition of the very young in the districts. The movement has been attended with the happiest results, and the mortality amongst the children has shown a very marked decrease. The method employed by the doctor-inspector and his assistants is to train and educate those who have the care of babies, more especially to follow ordinary rules of hygiene and dieting. The French Health Society has recognised Dr. Brallet's good offices by presenting him with a medal.

